# Host Differences in Influenza-Specific CD4 T Cell and B Cell Responses Are Modulated by Viral Strain and Route of Immunization

**DOI:** 10.1371/journal.pone.0034377

**Published:** 2012-03-23

**Authors:** Aarthi Sundararajan, Lifang Huan, Katherine A. Richards, Glendie Marcelin, Shabnam Alam, HyeMee Joo, Hongmei Yang, Richard J. Webby, David J. Topham, Andrea J. Sant, Mark Y. Sangster

**Affiliations:** 1 David H. Smith Center for Vaccine Biology and Immunology, Department of Microbiology and Immunology, University of Rochester Medical Center, Rochester, New York, United States of America; 2 Department of Microbiology, University of Tennessee, Knoxville, Tennessee, United States of America; 3 Department of Infectious Diseases, Division of Virology, St. Jude Children's Research Hospital, Memphis, Tennessee, United States of America; 4 Baylor Institute for Immunology Research, Baylor University Medical Center, Dallas, Texas, United States of America; 5 Department of Biostatistics and Computational Biology, University of Rochester Medical Center, Rochester, New York, United States of America; University of Iowa, United States of America

## Abstract

The antibody response to influenza infection is largely dependent on CD4 T cell help for B cells. Cognate signals and secreted factors provided by CD4 T cells drive B cell activation and regulate antibody isotype switching for optimal antiviral activity. Recently, we analyzed HLA-DR1 transgenic (DR1) mice and C57BL/10 (B10) mice after infection with influenza virus A/New Caledonia/20/99 (NC) and defined epitopes recognized by virus-specific CD4 T cells. Using this information in the current study, we demonstrate that the pattern of secretion of IL-2, IFN-γ, and IL-4 by CD4 T cells activated by NC infection is largely independent of epitope specificity and the magnitude of the epitope-specific response. Interestingly, however, the characteristics of the virus-specific CD4 T cell and the B cell response to NC infection differed in DR1 and B10 mice. The response in B10 mice featured predominantly IFN-γ-secreting CD4 T cells and strong IgG2b/IgG2c production. In contrast, in DR1 mice most CD4 T cells secreted IL-2 and IgG production was IgG1-biased. Infection of DR1 mice with influenza PR8 generated a response that was comparable to that in B10 mice, with predominantly IFN-γ-secreting CD4 T cells and greater numbers of IgG2c than IgG1 antibody-secreting cells. The response to intramuscular vaccination with inactivated NC was similar in DR1 and B10 mice; the majority of CD4 T cells secreted IL-2 and most IgG antibody-secreting cells produced IgG2b or IgG2c. Our findings identify inherent host influences on characteristics of the virus-specific CD4 T cell and B cell responses that are restricted to the lung environment. Furthermore, we show that these host influences are substantially modulated by the type of infecting virus via the early induction of innate factors. Our findings emphasize the importance of immunization strategy for demonstrating inherent host differences in CD4 T cell and B cell responses.

## Introduction

Studies of mouse models of influenza A virus infection have produced a comprehensive but as yet incomplete picture of disease pathogenesis and the innate and adaptive antiviral mechanisms that contribute to viral clearance and recovery. The initial phase of influenza virus replication in epithelial cells, local macrophages, and dendritic cells triggers the rapid release of a range of cytokines and chemokines with antiviral and pro-inflammatory activity [1], [2]. In addition to limiting viral replication in the respiratory tract, these processes are critical for the optimal activation of antigen-specific B and T cells and the development of adaptive immunity [3]. The ultimate elimination of infectious virus from the respiratory tract is dependent on B and T cells through mechanisms such as the destruction of virus-infected cells by infiltrating cytotoxic CD8 T cells and the antiviral activity of progressively increasing antibody (Ab) levels [4].

Optimal virus-specific Ab production by B cells following influenza infection is dependent on CD4 T cell help. Although some antiviral Abs can be generated in the absence of CD4 T cells, Ab production is substantially more vigorous and effective following collaborative interactions between CD4 T cells and B cells [5], [6]. CD4 T cells provide cognate signals and secreted factors that drive B cell activation and differentiation and regulate Ab isotype switching. After cognate interactions of peptide:MHC class II (MHC II)-bearing B cells with CD4 T cells, activated B cells may differentiate via the extrafollicular pathway to rapidly generate a population of short-lived virus-specific Ab-secreting cells (ASCs), or they may enter B cell follicles and initiate germinal center (GC) reactions where long-lasting populations of ASCs and memory B cells expressing high affinity antiviral Abs are formed [7]. The progression of B cells through the GC reaction is dependent on a second phase of a cognate T cell help delivered by T follicular helper (Tfh) cells [8]. The CD4 T cell response to influenza infection has long been regarded as “Th1-polarized” and characterized by high levels of IL-2 and interferon (IFN)-γ secretion [9], [10]. A Th1-type cytokine profile fits well with the typical influenza-specific B cell response, which includes a predominance of the IgG2a (IgG2c in some mouse strains) and IgG2b isotypes. IFN-γ promotes the expression of IgG2a/IgG2c and IgG2b by B cells [11], [12].

Recently, we used HLA-DR1 transgenic (DR1) mice to define HLA-DR1-restricted epitopes recognized by influenza virus-specific CD4 T cells [13], [14]. DR1 mice were infected with the H1N1 influenza virus A/New Caledonia/20/99 (NC) and multiple strong epitopes were identified in the 5 viral proteins analyzed, HA, NA, NP, M1, and NS1. IL-2 production and cytokine ELISpot assays were used for identification of specific CD4 T cells. The current study was initiated to relate the specificity and frequency of CD4 T cells induced by NC infection in DR1 mice with the secretion of IFN-γ and IL-4, as well as IL-2. Surprisingly, the pattern of secreted cytokines was inconsistent with the expected Th1-polarized response and included a relatively high proportion of IL-4-secretors among the specific CD4 T cells. This prompted us to examine a conventional mouse strain with a defined NC-specific CD4 T cell repertoire and to conduct a parallel analysis of the influenza-specific B cell response. Our findings indicate that host factors modulate influenza-specific CD4 T cell and B cell responses in a lung-restricted fashion. In addition, we show that these lung-specific differences can be overridden by the type of infecting virus via early effects on the priming environment.

## Materials and Methods

### Ethics Statement

Experiments involving animals were performed in accordance with the recommendations in the Guide for the Care and Use of Laboratory Animals of the National Institutes of Health and with the approval of Animal Care and Use Committees at the University of Rochester (protocol numbers 2006-029, 2006-030, and 2008-023) and the University of Tennessee (protocol number 1283).

### Viruses

Infectious stocks of the following viruses were grown and titrated as previously described: (i) human influenza virus A/New Caledonia/20/99 (H1N1) [13], (ii) influenza A/Puerto Rico/8/34 (H1N1) (PR8) and A/HK/X31 (H3N2) (X31) [15], and (iii) murine gammaherpesvirus 68 (MHV68) [16]. Purified viruses for use in immunoassays were prepared by differential centrifugation and sucrose banding of virus stocks. Purified PR8 and X31 purchased from Charles River (Wilmington, MA) were used in some experiments. Influenza NC for use as an immunogen was inactivated by β-propiolactone treatment of stock virus prior to purification and exposure to UV light after purification. Complete inactivation was confirmed by absence of cytopathic effect in Vero cell monolayers inoculated with the treated virus. Viral protein concentrations were determined using the Bio-Rad protein assay (Bio-Rad, Hercules, CA). All virus preparations were stored at −80°C.

### Mice

HLA-DR1 transgenic mice (B10.M/J-TgN-DR1) [17] were obtained from D. Zaller (Merck) through Taconic laboratories and C57BL/10J (B10) mice were purchased from The Jackson Laboratory (Bar Harbor, ME). Mice were maintained under specific pathogen-free conditions and were used at 8–16 wk of age. Male and female mice were used for the analysis of CD4 T cell responses following influenza infection and for the measurement of influenza titers in the lung; otherwise, female mice were used in all experiments.

### Immunizations and Sampling

Mice were anesthetized with Avertin (2,2,2-tribromoethanol) given intraperitoneally before all immunizations. Infectious virus for intranasal (i.n.) inoculation was diluted in Dulbecco's PBS and a 30 µl volume was applied to the external nares. For intramuscular (i.m.) immunization, a total dose of 20 µg of inactivated virus was given in two injections, each of 10 µg (inoculum volume of 50 µl in PBS), into the tibialis anterior muscle of each leg. A plastic sleeve over the needle controlled the depth of injection.

Anesthetized mice were exsanguinated via the retro-orbital plexus before tissue sampling. Lymph nodes and spleen were collected and gently disrupted between the frosted ends of microscope slides to generate single-cell suspensions. Red blood cells were removed from spleen preparations by ammonium chloride lysis. Lungs to be titrated for infectious virus were homogenized in 1 ml HBSS containing antibiotics and 0.1% BSA. Homogenates were clarified by centrifugation, and supernatants were stored at −80°C. Lungs for the measurement of tissue levels of cytokines and chemokines were perfused with ice-cold PBS before removal and were processed as previously described [18]. Briefly, lungs were homogenized in T-PER Tissue Protein Extraction Reagent (Pierce, Rockford, IL) containing Complete Mini Protease Inhibitor Cocktail tablets (Roche, Indianapolis, IN). Homogenates were centrifuged and the supernatants were stored at −80°C.

### Flow Cytometry for B Cell Phenotyping

Cell suspensions were stained with the following directly conjugated reagents at previously determined optimal concentrations: anti-CD4/CD8 PE-Cy7 (RM4-5/53-6.7), anti-CD45R/B220 PerCP (RA3-6B2), anti-CD95/Fas Alexa 647 (Jo2), and anti-CD138 allophycocyanin (281-2), (BD Biosciences), anti-CD19 PE-Cy5.5 (1D3) (eBioscience), and PNA FITC (Vector Laboratories). Live/dead fixable violet staining kit (Invitrogen) was used to discriminate dead cells. Data were acquired using an LSR II flow cytometer (BD Biosciences) and analyzed using FlowJo software (TreeStar). ASCs were defined as CD4^−^ CD8^−^ CD19^+^ B220^int^ CD138^+^ cells [19]; GC B cells were defined as CD4^−^ CD8^−^ B220^+^ PNA^+^ Fas^+^ cells [20].

### ELISpot Assay for Cytokine-Secreting Cells

Cytokine production by CD4 T cells was analyzed by ELISpot assay as previously described [13], [21]. Briefly, 96-well filter plates were coated with 2 µg/ml purified rat anti-mouse IL-2, IFN-γ, or IL-4 (clones JES6-1A12, AN18, and 11B11 respectively; BD Biosciences), then washed and blocked. CD4 T cells enriched by negative selection from the pooled tissues of 3–5 mice were plated at a range of concentrations (typically 50,000–300,000 cells/well), together with DAP-3 fibroblasts expressing the HLA-DR1 MHC class II protein (35,000 cells/well) or with T cell-depleted syngenic splenocytes (500,000 cells/well). Selected peptides or peptide pools were included in the cultures (10 µM of each peptide/well) and plates were incubated for 18–20 hr at 37°C with 5% CO_2_. Spots representing cytokine-secreting cells were visualized by developing the plates, in sequence, with biotinylated rat anti-mouse cytokine Abs (clones JES6-5H4 (IL-2), XMG1.2 (IFN-γ) and BVD6-24G2 (IL-4); BD Biosciences), alkaline phosphatase-conjugated streptavidin, and alkaline phosphatase substrate. A previously defined immunodominant peptide (HA-75) was included in most ELISpot assays to control for the degree of CD4 T cell priming. Peptides used in the ELISpot assay were obtained from the NIH/NIAID Biodefense and Emerging Infections Research Repository and were handled as previously described [13].

### ELISpot Assay for Ab-Secreting Cells

Virus-specific ASCs were enumerated by ELISpot assay as previously described [22]. Briefly, plates were coated with purified virus and single-cell suspensions were plated and incubated. Alkaline phosphatase-conjugated goat anti-mouse Abs with specificity for immunoglobulin (Ig) isotypes (Southern Biotechnology, Birmingham, AL) were used in combination with the substrate 5-bromo-4-chloro-3-indolyl phosphate to generate spots.

### ELISA

Virus-specific Ab levels in sera were determined by ELISA as previously described [16]. Briefly, serial 3-fold sample dilutions were added to virus-coated plates, and bound Ab was detected with alkaline phosphatase-conjugated goat anti-mouse Abs with specificity for Ig isotypes or IgG (Southern Biotechnology) and *p*-nitrophenyl phosphate substrate. The virus-specific serum Ab titer is expressed as the reciprocal of the highest dilution giving an absorbance value more than twice that for simultaneously titrated samples from naïve mice.

Total serum levels of IgM, IgG1, IgG2b, IgG2c, IgG3, and IgA were determined by a sandwich ELISA [16]. Concentrations were calculated from curves constructed using purified mouse Ig standards (Southern Biotechnology).

### Virus Titration

Viral titers in lung homogenates were determined by 50% tissue culture infective dose assay using Madin Darby canine kidney cells [23].

### CD4 T Cell Depletion

Mice were depleted of CD4 T cells by intraperitoneal administration of 200 µg of the CD4 T cell-specific mAb GK1.5 twice before and twice after infection. Non-depleted control mice were given an isotype-matched mAb.

### Multiplex and ELISA for Lung Cytokines and Chemokines

Lung homogenate supernatants were assayed for a panel of cytokines and chemokines using a 32-plex multiplex kit (Millipore) according to the manufacturer's instructions. The concentration of IFN-α was measured using a commercially available ELISA kit (PBL, Piscataway, NJ).

### Statistical Analysis

Cytokine and chemokine concentrations in lung homogenates were analyzed by two-way ANOVA after log transformation to stabilize variance, followed by the Tukey-Kramer correction to control type I error. Other comparisons of group means were performed using a two-tailed Student's *t*-test or nonparametric Mann–Whitney *U*-test for unpaired samples. Statistically significant P values are indicated by one (P<0.05), two (P<0.01), or three (P<0.001) asterisks.

## Results

### Contrasting patterns of CD4 T cell cytokine production in DR1 and B10 mice following NC infection

**Figure 1:**
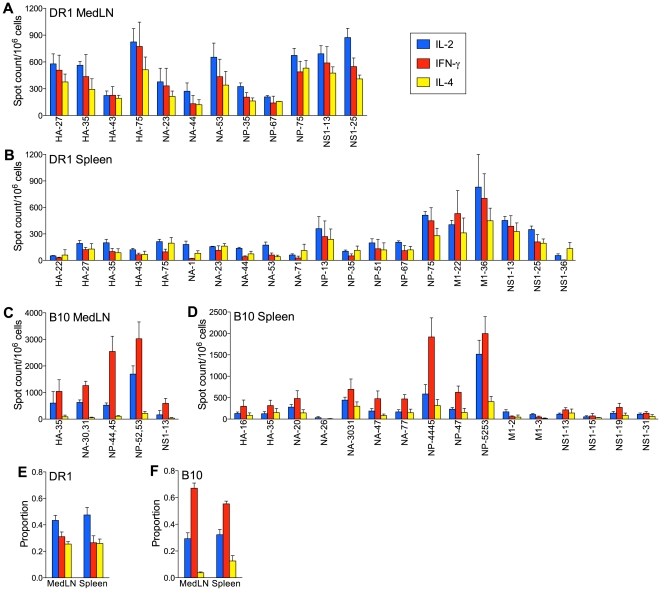
The CD4 T cell response to NC infection. (A–D) Cytokine production by peptide-specific CD4 T cells. DR1 (A and B) and B10 (C and D) mice were infected intranasally with 40,000 EID_50_ NC. Enriched CD4 T cells from the MedLN and spleen were analyzed on day 10 after infection. Frequencies of CD4 T cells secreting IL-2, IFN-γ, or IL-4 were determined by ELISpot assay after in vitro stimulation with antigen-presenting cells and individual 17-mer peptides. Peptide designations (x-axis) include the viral proteins of origin (HA, NA, NP, M1, and NS1). Results are normalized to spot counts per 10^6^ CD4 T cells and are shown as the mean+SEM for 2–6 independent experiments for each peptide. Cells from at least three mice were pooled for each experiment. (E, F) Proportions of peptide-specific CD4 T cells secreting IL-2, IFN-γ, or IL-4. Results are compiled from the data shown in A–D and represent 4 (MedLN) or 6 (spleen) independent experiments evaluating 3–12 (MedLN) or 12–20 (spleen) individual peptides. The mean+SEM is shown.

In our analysis of the specificity of CD4 T cells after influenza NC infection in DR1 mice, we identified a broad CD4 T cell repertoire, with recognition of multiple HLA-DR1-restricted epitopes in each of the HA, NA, NP, M1, and NS1 viral proteins. This information enabled us to now ask whether the expected Th1-type profile of secreted cytokines after influenza infection was influenced by T cell epitope specificity. CD4 T cells purified from the mediastinal lymph node (MedLN) and spleen on day 10 after NC infection of DR1 mice were tested for their ability to secrete IL-2, IFN-γ, or IL-4 after stimulation with defined peptide epitopes. The patterns of secretion of these cytokines by peptide-reactive CD4 T cells were similar in the MedLN and spleen and largely independent of peptide specificity and the magnitude of the peptide-specific response (Fig. 1, A and B). Generally, within each peptide-specific CD4 T cell population a slightly larger proportion of the cells produced IL-2 and similar proportions produced IFN-γ or IL-4.

The comparable proportions of IL-4- and IFN-γ-secretors among the virus-specific CD4 T cells in DR1 mice was surprising and inconsistent with the Th1-polarized response expected after influenza infection. We therefore conducted a similar analysis in B10 mice, a conventional mouse strain in which we had analyzed the response to NC infection and defined CD4 T cell-recognized epitopes in same set of viral proteins analyzed in DR1 mice [21]. B10 mice share the non-MHC genetic background of DR1 mice. As was the case in DR1 mice, the pattern of cytokine production by virus-specific CD4 T cells in B10 mice was independent of epitope specificity and response magnitude (Fig. 1, C and D). However, in contrast to the result in DR1 mice, IFN-γ-secreting cells clearly predominated over IL-2-secreting cells in B10 mice and IL-4-secreting cells formed a relatively small proportion. The contrasting patterns of cytokine secretion in DR1 and B10 mice are summarized in Fig. 1, E and F, which show the proportions of IL-2-, IFN-γ-, and IL-4-secretors among combined peptide-specific CD4 T cells.

### The B cell response to NC infection in DR1 mice is delayed and IgG1-biased

**Figure 2:**
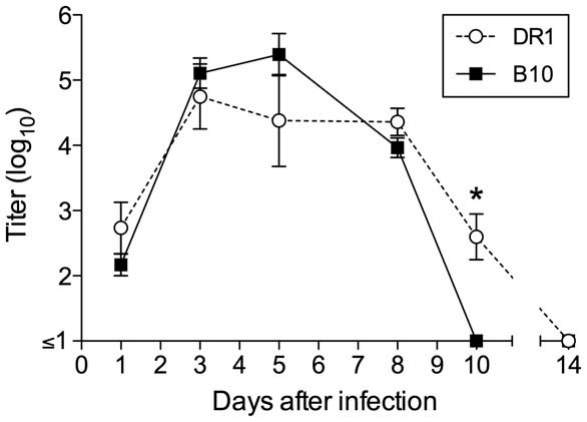
Replication of NC in the lung. DR1 and B10 mice were sampled at intervals after intranasal infection. Virus titers are represented as log_10_ TCID_50_/0.2 ml of lung homogenate. The mean ± SE is shown for 3–4 (days 1, 3, 5, and 14) and 10–20 (days 8 and 10) individual mice per group. * P<0.05.

**Figure 3:**
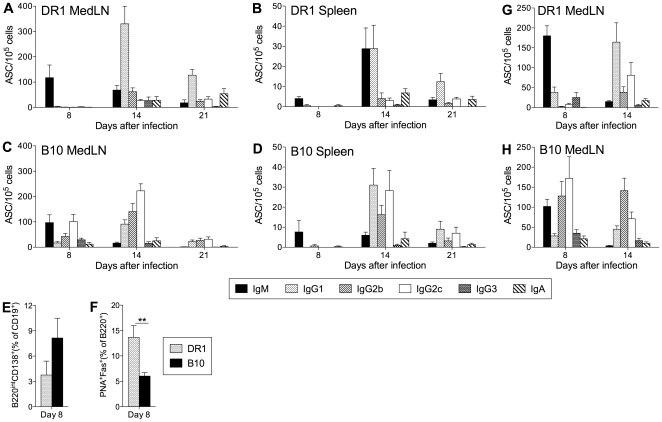
The B cell response to NC infection. (A–D) Virus-specific ASC frequencies. DR1 (A and B) and B10 (C and D) mice were infected intranasally with 40,000 EID_50_ NC. Virus-specific ASC frequencies in the MedLN and spleen were determined by ELISpot assay at intervals after infection. (E, F) Flow cytometric analysis of ASCs and germinal center B cells. MedLN cells were analyzed on day 8 after intranasal NC infection (40,000 EID_50_ dose) of DR1 and B10 mice. ASC frequencies (E) represent the proportion of B220^int^ CD138^+^ cells after gating on live CD4^−^ CD8^−^ CD19^+^ cells. Germinal center B cell frequencies (F) represent the proportion of PNA^+^ Fas^+^ cells among live CD4^−^ CD8^−^ B220^+^ cells. Representative staining profiles are shown in figure S1. (G, H) Virus-specific ASC frequencies in the MedLN of DR1 and B10 mice after intranasal infection with a high dose (100,000 EID_50_) of NC. Fig. 3 data sets depict the mean+SE for 3–8 individual mice per group. ** P<0.01.

The contrasting patterns of cytokine production by virus-specific CD4 T cells in DR1 and B10 mice after NC infection prompted us to investigate B cell response differences, since cytokines produced by T cells during cognate interactions with B cells play a key role in directing the expression of particular Ig isotypes. In both DR1 and B10 mice, NC infection was sublethal and infectious virus was eliminated from the lung after a clear phase of replication. Virus titers in the lung on days 1, 3, 5, and 8 after infection were similar in DR1 and B10 mice, indicating comparable antigen loads (Fig. 2). By day 8, a strong virus-specific IgM and IgG ASC response had developed in the draining MedLN of B10 mice, but IgM ASCs predominated in the MedLN of DR1 mice (Fig. 3, A, B, C, D). The response in the MedLN of B10 mice was typical of previously described influenza-specific B cell responses in several mouse strains and included a strong IgG2b and IgG2c component. When a NC-specific IgG response developed in DR1 mice, it was strongly biased to IgG1 production (see MedLN and spleen on day 14). The percentage of CD19^+^ B cells in the MedLN on day 8 was similar in DR1 and B10 mice. There was a trend towards a higher proportion of CD19^+^ cells with the B220^int^ CD138^+^ phenotype of ASCs in B10 mice, but the difference from DR1 mice was not significant (Fig. 3E and Fig. S1). The proportion of GC cells (PNA^+^ Fas^+^) in the B220^+^ cell population was significantly higher in DR1 mice (Fig. 3F and Fig. S1), suggesting stronger GC activity. Infection with a higher dose of NC did not substantially modify the differences between DR1 and B10 mice in the features of the NC-specific ASC response in the MedLN (Fig. 3, G and H). We concluded that the different patterns of Ig isotype expression in DR1 and B10 mice after NC infection fit closely with the contrasting patterns of cytokine production by virus-specific CD4 T cells, since IL-4 and IFN-γ act reciprocally in promoting expression of IgG1 and IgG2b/IgG2c, respectively [12].

**Figure 4:**
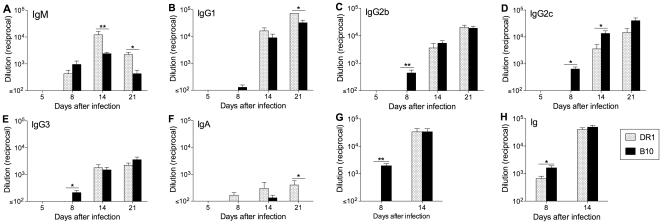
Serum levels of virus-specific Ab in DR1 and B10 mice after NC infection. NC-specific IgM (A), IgG1 (B), IgG2b (C), IgG2c (D), IgG3 (E), IgA (F), IgG (G), and Ig (H) titers were determined by ELISA on plates coated with disrupted viral particles. Titers are shown as the reciprocal of the highest serum dilution scored as positive relative to naïve control serum. The mean+SE is shown for 4–8 individual mice per group. * P<0.05, ** P<0.01.

ELISA measurements of NC-specific serum Abs were consistent with the pattern of ASC generation in lymphoid tissues (Fig. 4). Early virus-specific IgG levels were significantly higher in B10 compared with DR1 mice, primarily reflecting increased IgG2b, IgG2c, and IgG3 production. IgM levels did not follow this pattern and were significantly higher in DR1 mice on days 14 and 21. NC-specific serum Ig levels were significantly higher in B10 compared with DR1 mice on day 8 and similar at later sampling times.

### The quality of the NC-specific B cell response in DR1 mice depends on the form of immunization

**Figure 5:**
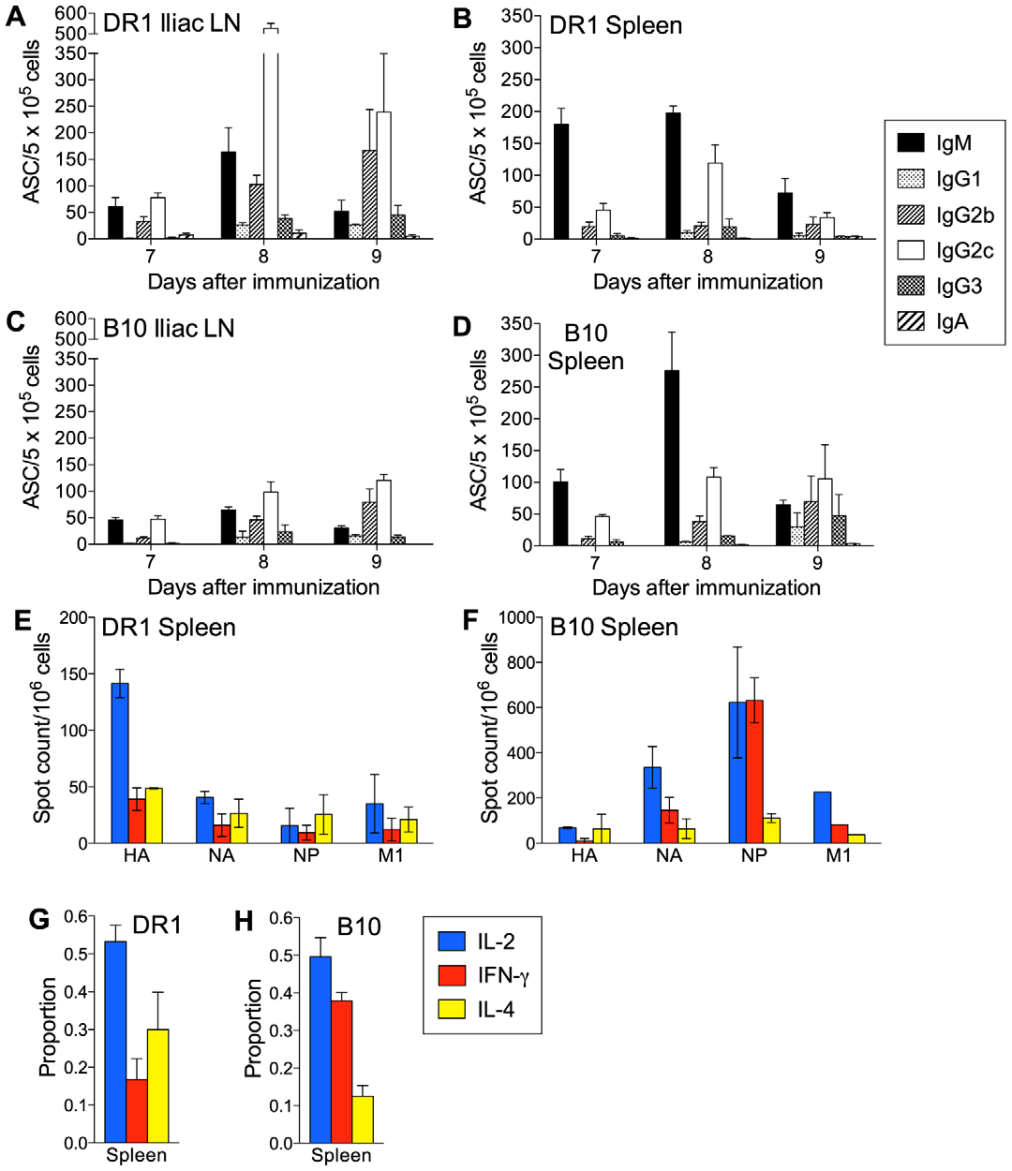
The virus-specific B cell and CD4 T cell response to intramuscular immunization. (A–D) Virus-specific ASC frequencies. DR1 (A and B) and B10 (C and D) mice were immunized intramuscularly with inactivated NC. Virus-specific ASC frequencies in the iliac lymph nodes and spleen were determined by ELISpot assay at the indicated times after immunization. The mean+SE is shown for 3–4 individual mice per group. (E, F) Cytokine production by peptide-specific CD4 T cells. Enriched CD4 T cells from the spleen were analyzed on day 8 after immunization. Frequencies of CD4 T cells secreting IL-2, IFN-γ, or IL-4 were determined by ELISpot assay after in vitro stimulation with antigen-presenting cells and sets of 1–3 17-mer peptides from different viral proteins (HA, NA, NP, and M1). Results are normalized to spot counts per 10^6^ CD4 T cells and are shown as the mean ± range for 2 independent experiments (the M1 protein was represented in only one experiment with B10 mice). Cells from at least three mice were pooled for each experiment. (G, H) Proportions of peptide-specific CD4 T cells secreting IL-2, IFN-γ, or IL-4. Results are compiled from the data shown in E and F. The mean ± range is shown for the two independent experiments.

Total serum levels of IgM and the IgG isotypes were similar in uninfected DR1 and B10 mice (Fig. S2), suggesting that the prominence of IgG1 in the response of DR1 mice to NC infection did not reflect an intrinsic bias impacting B cell responses in general. We therefore compared B cell and CD4 T cell responses in DR1 and B10 mice following i.m. administration of purified inactivated NC virus particles, a strategy that allowed us to compare CD4 T cell responses to the same epitopes after a different mode of immunization. Virus-specific IgG responses were largely comparable in DR1 and B10 mice; the responses followed similar kinetics and were characterized by a predominance of the IgG2b/IgG2c isotypes (Fig. 5, A, B, C, D). Not only was there no IgG1 bias in the response in DR1 mice, the IgG2c response in the IliLN was generally more vigorous in DR1 than in B10 mice.

In both DR1 and B10 mice after i.m. immunization, the highest proportion of peptide-specific CD4 T cells secreted IL-2, with smaller proportions secreting IFN-γ- or IL-4 (Fig. 5, E, F, G, H). Our findings point to the lung environment as a key element in the delay and IgG1 bias of the IgG response to NC infection in DR1 mice. Furthermore, they indicate that a predominance of IFN-γ-secreting cells is not a prerequisite for an IgG2b/IgG2c-biased B cell response.

### The virus-specific B cell and CD4 T cell response to lung infection in DR1 mice is modulated by the infecting virus

**Figure 6:**
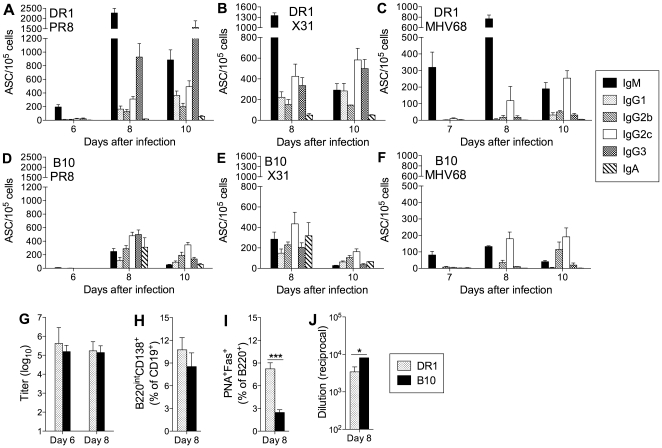
The B cell response following infection with different viruses that replicate in the lung. (A–F) Virus-specific ASC frequencies. DR1 (A–C) and B10 (D–F) mice were infected intranasally with the influenza viruses PR8 (A and D) and X31 (B and E), and with the non-influenza virus MHV68 (C and F). Virus-specific ASC frequencies in the MedLN were determined by ELISPOT assay at intervals after infection. (G) PR8 replication in the lung. Titers are represented as log_10_ TCID_50_/0.2 ml of lung homogenate. (H, I) Flow cytometric analysis of ASCs and germinal center B cells in the MedLN on day 8 after PR8 infection. ASC frequencies (H) represent the proportion of B220^int^ CD138^+^ cells after gating on live CD4^−^ CD8^−^ CD19^+^ cells. Germinal center B cell frequencies (I) represent the proportion of PNA^+^ Fas^+^ cells among live CD4^−^ CD8^−^ B220^+^ cells. (J) Serum levels of virus-specific IgG in DR1 and B10 mice on day 8 after PR8 infection. Titers determined by ELISA are shown as the reciprocal of the highest serum dilution scored as positive relative to naïve control serum. Fig. 6 data sets depict the mean+SE for 3–10 individual mice per group. * P<0.05, *** P<0.001.

To determine whether the response to NC infection in DR1 mice was independent of virus type, we compared B cell responses in DR1 and B10 mice after infection with other influenza viruses (PR8 and X31), and with a non-influenza virus (MHV68) that also replicates in the lung. In contrast to what was observed after NC infection, infection of DR1 mice with PR8, X31, or MHV68 generated a virus-specific B cell response in the MedLN that was largely comparable to the response in B10 mice (Fig. 6, A, B, C, D, E, F). Most notably, the IgG response of DR1 mice was not biased to IgG1 production. Interestingly, the early IgM ASC response was stronger in DR1 compared with B10 mice after PR8, X31, and MHV68 infection, a trend that was also evident after NC infection.

The response to PR8 infection in DR1 and B10 mice was compared in greater detail. Virus titers in the lungs were similar on days 6 and 8, indicating similar viral loads (Fig. 6G). Flow cytometric analysis of MedLN cells on day 8 showed similar percentages of CD19^+^ B cells and similar proportions of CD19^+^ cells that were B220^int^ CD138^+^ (Fig. 6H), indicating comparable early ASC generation. The proportion of GC cells (PNA^+^ Fas^+^) in the B220^+^ cell population was significantly higher in DR1 compared with B10 mice (Fig. 6I), as was also the case after NC infection, suggesting an inherent tendency towards a stronger GC response in DR1 mice regardless of the infecting virus. Early serum levels of PR8-specific IgG were significantly lower in DR1 than in B10 mice (Fig. 6J), consistent with the situation after NC infection. Thus, modulation of the IgG isotype expression profile in DR1 mice by PR8 infection was not associated with an increased rate of IgG production. Although the high frequency of IgM ASCs in DR1 mice after PR8 infection raised the possibility of an unusually strong T-independent Ab response to this virus [24], we found that the formation of both IgM and IgG ASCs specific for PR8 in the MedLN of DR1 mice on day 8 was largely eliminated by CD4 T cell depletion (Fig. S3), indicating a dependence on CD4 T cell help.

**Figure 7:**
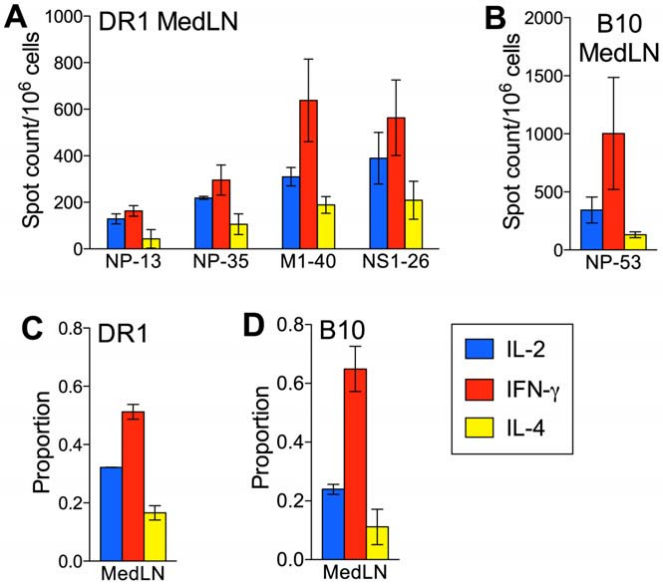
The CD4 T cell response to PR8 infection. (A, B) Cytokine production by peptide-specific CD4 T cells. DR1 and B10 mice were infected intranasally with PR8. Enriched CD4 T cells from the MedLN were analyzed on day 10 after infection. Frequencies of CD4 T cells secreting IL-2, IFN-γ, or IL-4 were determined by ELISpot assay after in vitro stimulation with antigen-presenting cells and individual 17-mer peptides. Peptide designations (x-axis) include the viral proteins of origin (NP, M1, and NS1). Results are normalized to spot counts per 10^6^ CD4 T cells and are shown as the mean ± range for 2 independent experiments. Cells from at least three mice were pooled for each experiment. (C, D) Proportions of peptide-specific CD4 T cells secreting IL-2, IFN-γ, or IL-4. Results are compiled from the data shown in A and B. The mean ± range is shown for the two independent experiments.

We next asked how the quality of the B cell response after PR8 infection related to the pattern of cytokines produced by virus-specific CD4 T cells. Purified CD4 T cells from the MedLN on day 10 after PR8 infection were tested for their ability to produce cytokines after stimulation with peptides known to be strong epitopes in NC and conserved between NC and PR8. In contrast to the response to NC infection, IFN-γ-secreting cells were consistently more frequent than IL-2-secreting cells in DR1 mice after PR8 infection (Fig. 7, A and C). The profile in B10 mice after PR8 infection was similar to that after NC infection, with clearly the majority of virus-specific CD4 T cells secreting IFN-γ (Fig. 7, B and D). Our findings demonstrate that the type of infecting virus modulates the virus-specific B cell response to infection of the respiratory tract in DR1 mice, perhaps via an increase in the proportion of IFN-γ-secreting CD4 T cells.

### Antigen-independent effects of lung infection modulate the virus-specific B cell response in DR1 mice

**Figure 8:**
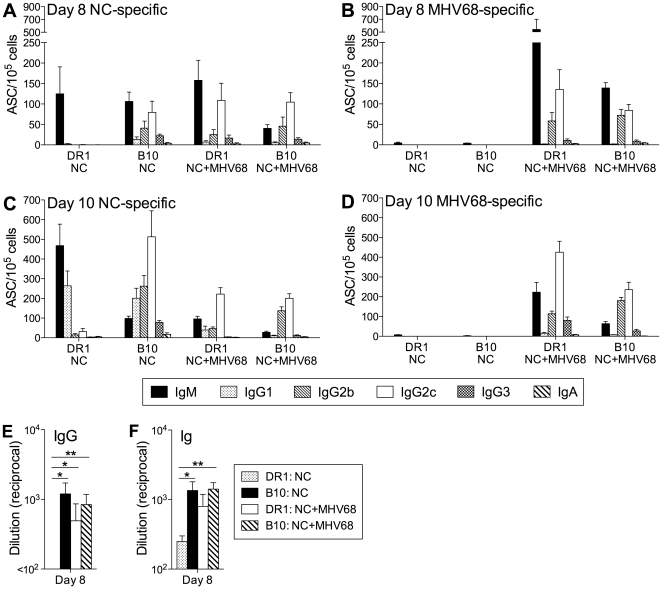
The NC-specific B cell response in DR1 mice is modulated by concurrent infection with MHV68. (A–D) Virus-specific ASC frequencies. DR1 and B10 mice were infected intranasally with NC only, or with NC and MHV68. ASC frequencies in the MedLN were determined by ELISPOT assay on day 8 (A and B) and day 10 (C and D) after infection. ASCs specific for NC or MHV68 were detected using plates coated with disrupted NC (A and C) and disrupted MHV68 (B and D), respectively. (E, F) NC-specific serum IgG (E) and Ig (F) levels. Titers were determined by ELISA and are shown as the reciprocal of the highest serum dilution scored as positive relative to naïve control serum. The mean+SE is shown for 4–9 individual mice per group. * P<0.05, ** P<0.01.

We hypothesized that the contrasting B cell responses in DR1 mice following infection with NC compared with PR8 and the other viruses tested reflected differences in the early induction of innate factors that set the character of the immune response. To test this idea, we asked whether the response to NC infection would be modified by concurrent MHV68 infection. MHV68 was selected for this experiment because it is not cross-reactive with influenza virus at the B cell level [16] and it elicits a B cell response in DR1 mice with a clear IgG2c predominance among switched Ab isotypes (Fig. 6C). Cohorts of DR1 and B10 mice were infected with NC plus MHV68, or with NC only, and the B cell response on days 8 and 10 was assessed by ELISpot assay using plates coated with NC or MHV68 (Fig. 8, A, B, C, D). The NC-specific response in DR1 mice infected with NC alone was modified by concurrent MHV68 infection and resembled the response in B10 mice infected with NC alone. Notably, the NC-specific IgG response in co-infected DR1 mice was not delayed relative to the response in B10 mice and the predominant IgG isotype was IgG2c, not IgG1. Serum levels of NC-specific IgG and Ig on day 8 were also consistent with a more rapid NC-specific response in co-infected DR1 mice compared with those infected with NC alone (Fig. 8, E and F). This experiment demonstrates that the IgG1 bias of a B cell response to infection in DR1 mice can be overridden by bystander influences of events taking place in the same tissue.

### Virus type and host influence innate responses to influenza infection

**Figure 9:**
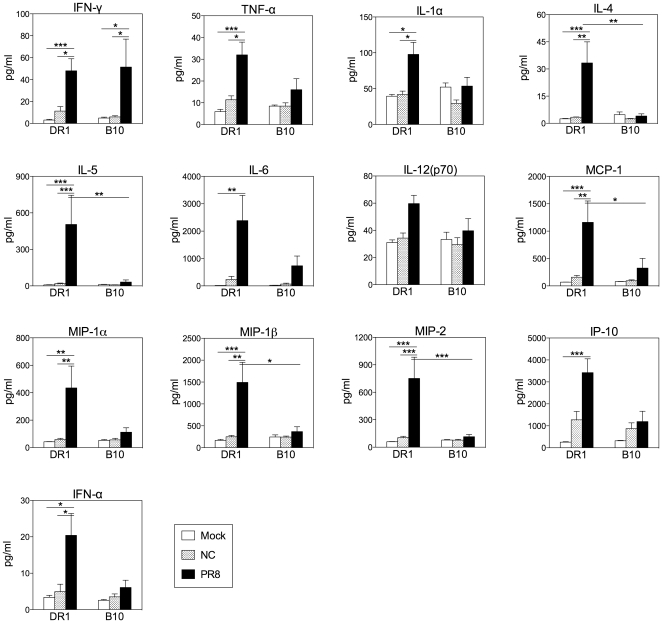
Cytokine and chemokine production in the lung after influenza infection. DR1 and B10 mice were infected intranasally with NC or PR8 or were mock-infected with PBS. Mice were sampled 60 h after inoculation. Cytokine and chemokine concentrations in clarified lung homogenates were determined by Multiplex assay or by sandwich ELISA (IFN-α only). Figure 9 shows the results for a selection of the 30 cytokine and chemokine determinations. Results for the remaining cytokines and chemokines are shown in figure S4. The mean+SE is shown for 5 individual mice per group. * P<0.05, ** P<0.01, *** P<0.001.

To explore whether any differences in innate responses could be detected in DR1 and B10 mice, we measured the levels of a large range of cytokines and chemokines in the lungs of DR1 and B10 mice 60 h after infection with influenza NC or PR8 (Fig. 9 and Fig. S4). We speculated that the levels of proinflammatory and Th1-polarizing factors would be lower in DR1 compared with B10 mice after NC infection. However, this idea was not supported by our results. After NC infection, there was a trend towards higher levels of several cytokines and chemokines in DR1 than in B10 mice (IFN-γ and IL-6 for example), but differences were not statistically significant. Thus, we found no evidence for inherent differences in early innate responses after NC infection of DR1 and B10 mice that would explain the distinct patterns of CD4 T cell cytokines and B cell responses. The clear trend after PR8 infection was a greater increase in the baseline levels of proinflammatory factors in DR1 than in B10 mice. IFN-γ was the only measured factor with a concentration that was significantly higher than baseline levels after PR8 infection of B10 mice, whereas the levels of multiple proinflammatory cytokines and chemokines (IFN-α, IFN-γ, TNF-α, IL-1α, IL-6, MCP-1, MIP-1β, MIP-2, and IP-10 for example) were significantly increased in DR1 mice. Interestingly, there were also significant increases in the concentrations of the Th2-type cytokines IL-4 and IL-5 in DR1 mice after PR8 infection. Taken together, our findings raise the possibility that the concentrations of proinflammatory factors in the lungs of DR1 mice after PR8 infection were sufficient to override influences that predominated after NC infection.

## Discussion

The current study was initiated to relate patterns of cytokine secretion by influenza-specific CD4 T cells to epitope specificity and the magnitude of epitope-specific responses. A comprehensive set of epitopes in DR1 and B10 mice were available to us, since we had recently analyzed these mice after infection with influenza NC and defined MHC II-restricted epitopes in a broad range of viral proteins [13], [14], [21]. The epitopes defined in DR1 mice were HLA-DR1-restricted, whereas those in B10 were restricted to I-A^b^, the only MHC II molecule expressed in this strain. DR1 mice represent an established transgenic system for defining HLA-DR1-restricted epitopes that may be relevant to human responses and for functional studies of the epitope-specific CD4 T cells [17], [25]. Antigen-presenting cells in DR1 mice express a transgene-encoded hybrid MHC II molecule consisting of the peptide-binding domain of human HLA-DR1 and a membrane-proximal region of mouse origin, a structure that permits normal interaction of the transgenic molecule with murine CD4 during T cell epitope recognition. The repertoire that we defined in DR1 mice was exceptionally broad and included multiple strong epitopes in all of the viral proteins examined (HA, NA, NP, M1, and NS1). In contrast, the repertoire in B10 mice was much narrower and comprised approximately one-fifth the number of epitopes in DR1 mice. Within the same set of viral proteins in the B10 study, strong or moderate epitopes were confined to the NP and NA. Both DR1 and B10 mice were evaluated in the current study, which screened epitope-specific CD4 T cells for the secretion of IL-2 (the cytokine used as readout during epitope identification), as well as IFN-γ and IL-4, the prototypic Th1- and Th2-type cytokines, respectively. An observation that was consistent in DR1 and B10 mice after NC infection was that the pattern of secretion of IL-2, IFN-γ, and IL-4 was independent of epitope specificity or whether epitopes were major or minor in terms of the number of stimulated CD4 T cells. Apparently, the priming environment is the overriding influence on the quality of the CD4 T cell response. What also emerged from our analysis after NC infection were differences in cytokine secretion patterns between DR1 and B10 mice. This prompted us to evaluate CD4 T cell and B cell responses in parallel because of the link between CD4 T cell-derived cytokines and the pattern of Ig isotype expression.

A clear majority among all epitope-specific CD4 T cells in the MedLN and spleen of B10 mice after NC infection secreted IFN-γ, consistent with previous studies and the generally accepted Th1 nature of the response to influenza infection [9], [10]. The MedLN differed from the spleen in having a higher ratio of IFN-γ-secreting to IL-4-secreting CD4 T cells. Our analysis does not identify whether epitope-specific CD4 T cells produced one or more of the cytokines tested. The majority of IL-2-secreting cells in a Th1-polarized response are also likely to secrete IFN-γ [26], [27]. Our observation of a marked predominance of IFN-γ-secreting over IL-2-secreting cells in the B10 response is consistent with an expected subset of IFN-γ-secreting cells that are IL-2-negative [26]. In all likelihood the IFN-γ-secreting and IL-4-secreting cells represent distinct populations [28]. IFN-γ is strongly associated with the expression of IgG2a/c and IgG2b by activated B cells, whereas IL-4 negatively regulates production of these isotypes and promotes IgG1 expression [12]. Thus, the cytokine secretion patterns fit well with the prominence of IgG2b and IgG2c in the NC-specific Ab response, especially in the MedLN, and the larger proportion of IgG1 ASCs in the spleen. It is unclear whether the different patterns of cytokine secretion by CD4 T cells in the MedLN compared with the spleen reflect different polarizing influences on cells activated in the two sites and/or differences between Th cell subsets in site-to-site trafficking after activation.

The pattern of cytokine production by epitope-specific CD4 T cells in DR1 mice after NC infection was surprising. In contrast to the Th1-polarized response and IFN-γ predominance in B10 mice, IL-4 secretion was prominent and comparable to IFN-γ secretion in both the MedLN and spleen of DR1 mice. In addition, the NC-specific ASC response in DR1 mice had unusual features and differed from what can be considered a typical B cell response as exemplified in B10 mice [6], [29]. Although early IgM ASC formation was similar in DR1 and B10 mice, the initial IgG2b/IgG2c response in B10 was essentially absent in DR1 mice. When an IgG response developed in DR1 mice, it was strongly IgG1-biased. The skewing towards IgG1 production in DR1 mice is consistent with the lower ratio of IFN-γ-secreting to IL-4-secreting CD4 T cells. This is further supported by the similarity between the IgG isotype distribution pattern in DR1 mice and the response to influenza in IFN-γ-deficient mouse models [30], [31]. The prominence of IL-4-secreting CD4 T cells in DR1 mice also suggests an explanation for the stronger GC reaction that we demonstrated in these mice compared with B10 mice. We did not measure Tfh cell frequencies in our analysis. However, there is evidence that IL-4 secretion in responding lymph nodes is largely restricted to Tfh cells [32], [33], suggesting that these cells may be more frequent in DR1 than in B10 mice. If this were the case, a stronger GC reaction in DR1 mice would fit with evidence for a direct relationship between the numbers of Tfh cells and GC B cells [34]. A recent study indicated that selection of B cells for entry into the GC reaction is favored by a high level of peptide:MHC II expression and the advantage conferred in competition for potentially limiting cognate T cell help [35]. Conceivably, T cell help to drive GC B cell formation may be less limiting in DR1 than in B10 mice because of the substantially broader CD4 T cell repertoire in DR1 mice [13], [14], [21]. This may contribute to the greater number of Tfh cells in DR1 mice, since Tfh cell formation is dependent on signals delivered during cognate T-B interactions.

The characteristics of the response to i.m. vaccination with inactivated NC differed from that following NC infection in both DR1 and B10 mice. In B10 mice, there was a shift in the predominant CD4 T cell-secreted cytokine from IFN-γ after infection to IL-2 after vaccination. This trend has been described for CD4 T cells generated by protein vaccines compared with infections [26], and likely reflects the influence of infection-associated innate signals and the induction of factors that drive Th1 polarization. Although the cytokine secretion pattern in DR1 mice after i.m. vaccination resembled that after infection, the B cell response differed markedly; notably, the delayed IgG response and IgG1 bias after infection was not present after vaccination. Strikingly, B cell responses after vaccination were largely indistinguishable in DR1 and B10 mice and featured a predominance of the IFN-γ-associated IgG2c among switched Ab isotypes. Our findings demonstrate that IL-2-biased CD4 T cell responses generated by protein vaccines can support B cell responses that are typically associated with IFN-γ-secreting Th1-polarized cells. In addition, they indicate that the differences between DR1 and B10 mice in responses to lung infection reflect mechanisms that play little role in responses to i.m. vaccination.

Infection with the influenza viruses PR8 and X31 and the non-influenza virus MHV68 modulated key characteristics of the B cell response to NC infection in DR1 mice. Perhaps most notably, the IgG1 bias that was present in DR1 mice after NC infection was not present and the distribution of IgG isotypes largely resembled that in B10 mice. This may reflect an increase in the ratio of IFN-γ-secreting to IL-4-secreting CD4 T cells, as we demonstrated in DR1 mice after infection with PR8 compared with NC. Our analysis of early cytokine and chemokine production in the lung clearly demonstrated the greater capacity of PR8 (compared with NC) to induce factors that promote a Th1-polarizing environment. This likely reflects viral virulence determinants in PR8, such as replicative capacity, cell tropism, and cytotoxic potential, and a high level of activation of innate responses via pattern recognition receptor signaling. We demonstrated that co-infection changed the bias of the IgG response to NC infection in DR1 mice from IgG1 to IgG2b/IgG2c, indicating that this was mediated by non-specifically acting factors in the priming environment. In addition to their influence on CD4 T cell responses, innate factors and pattern recognition receptor ligands may also modulate B cell responses through direct interactions with B cells that promote activation and isotype switching [19], [36], [37], [38].

Two aspects of the early IgG response differed between DR1 and B10 mice after both NC and PR8 infection; virus-specific IgG production was significantly delayed and the proportion of GC B cells was significantly higher in DR1 mice. These differences are apparently unrelated to changes in the CD4 T cell cytokine secretion patterns that we analyzed. Cognate T cell help is a prerequisite for B cell entry into the GC reaction [7]. There is evidence that isotype switching decisions by B cells may be made before entry into GCs and thus may reflect the cytokine milieu during cognate T-B interactions [39]. Our observations may therefore reflect (i) a greater tendency for cognate T-B interactions to drive GC differentiation of B cells in DR1 mice compared with B10 mice (as discussed above), and (ii) modulation of CD4 T cell-secreted cytokine patterns by the type of infecting virus. The delay in virus-specific IgG production in DR1 mice would be explained if B cells directed to express any of the IgG1, IgG2b, or IgG2c isotypes after cognate T-B interactions preferentially differentiated via the GC pathway instead of rapidly forming ASCs via the alternative extrafollicular pathway. Interestingly, although IgG production after PR8 infection was delayed in DR1 compared with B10 mice, the IgM response was more rapid and substantially stronger in the DR1 mice. This feature of the IgM response in DR1 mice was also evident after X31 and MHV68 infection, and occasionally and to a much lesser degree after NC infection. The characteristics of the IgM and IgG responses in DR1 mice are reflected in the markedly higher ratio of virus-specific IgM ASCs to switched isotype ASCs early in the response in DR1 compared with B10 mice for all of the viruses used in our analysis (Fig. S5).

The T cell-dependent extrafollicular pathway of B cell differentiation is thought to generate the initial wave of Ab produced in response to influenza infection [4]. The idea that this pathway is responsible for the vigorous early IgM response to PR8 infection in DR1 mice is supported by our demonstration that the response is T cell-dependent and is associated with the appearance of a population of cells with the phenotype of extrafollicular ASCs. Regulation of the extrafollicular pathway is not well understood, but there is evidence that it is driven, at least in part, by the activity of innate factors such as type I IFN and IL-12 as well as cytokines derived from activated T cells [7], [36], [40], [41]. Our analysis of lung cytokines and chemokines after infection demonstrated a general pattern of higher levels in DR1 than in B10 mice. In addition, the levels of a number of these factors were markedly higher in DR1 mice after PR8 than after NC infection. Although many of these factors have not been investigated in the context of extrafollicular responses, our observations raise the possibility that the extrafollicular arm of the B cell response is stronger in DR1 than in B10 mice, perhaps especially after infection with PR8, X31, and MHV68. Innate factors may also enhance IgM production [42], resulting in an IgM bias in extrafollicular Ab production. A GC contribution to the early IgM response cannot be excluded, since GC B cells had developed in the MedLN of PR8-infected DR1 mice at the time of peak IgM ASC numbers.

Our understanding of the B cell response differences between DR1 and B10 mice is incomplete. Overall, our findings indicate that these differences are restricted to responses in the respiratory tract and are modulated (but not eliminated) by the type of infecting virus through effects on the quality of the CD4 T cell response. The difference between DR1 and B10 mice was most distinct after NC infection; DR1 and B10 mice were clearly different at the level of CD4 T cell cytokine secretion patterns and the profile of IgG isotype expression after NC infection, but not after PR8 infection. Although our data are consistent with a difference that is imparted at the initiation of the response in the lung, our analysis of early cytokine and chemokine production in the lungs after NC infection did not suggest basis for this difference. There is evidence that antigen-presenting cells can be programmed to bias CD4 T cell responses toward the Th2 pathway of differentiation [43]. An antigen-presenting cell subset termed late-activator antigen-presenting cells or LAPCs have been shown to migrate from the lung to the MedLN during influenza infection and selectively induce Th2 responses [44]. It will be of interest to evaluate the activity of LAPCs during responses to different viruses and in different mouse strains.

In summary, our analysis demonstrates that the pattern of secretion of IL-2, IFN-γ, and IL-4 by activated CD4 T cells is largely independent of epitope specificity and the magnitude of the epitope-specific response. Rather, the more important influences on these responses are the form of immunization and inherent host characteristics. In addition, we demonstrate that CD4 T cell responses to infection of the respiratory tract are substantially modulated by the type of infecting virus in a manner that is host-dependent and reflects changes in the early induction of innate factors. What also emerges from our analysis is the DR1 mouse as a potentially useful model to gain insights into regulation of the B cell response to viral infection of the respiratory tract. An unexpected but important insight derived from our work is the importance of immunization strategy for revealing inherent host differences in CD4 T cell and B cell responses.

## Supporting Information

Figure S1 **Flow cytometric identification of ASCs and germinal center B cells.** MedLN cells were analyzed on day 8 after intranasal NC infection of DR1 and B10 mice. Representative 5% contour plots are shown for individual DR1 and B10 mice after infection and for naïve control mice. Plots depict B220 and CD138 expression after gating on live CD4^−^ CD8^−^ CD19^+^ cells (A) or PNA-binding and Fas expression after gating on live CD4^−^ CD8^−^ B220^+^ cells (B). Numbers indicate cell frequencies in the depicted gates identifying ASCs (defined as B220^int^ CD138^+^) and germinal center B cells (defined as PNA^+^ Fas^+^).
(TIF)

Figure S2 **Total serum levels of Ab isotypes in uninfected DR1 and B10 mice.** Titers were determined by ELISA and quantified by reference to standards of known concentration. The mean+SE is shown for 5 individual mice per group.
(TIFF)

Figure S3 **The virus-specific B cell response to PR8 infection in DR1 mice is CD4 T cell-dependent.** PR8-specific ASC frequencies in the MedLN of CD4 T cell-depleted DR1 mice and mock-depleted control mice were determined by ELISpot assay on day 8 after infection. The mean+SE is shown for 4 individual mice per group.
(TIFF)

Figure S4 **Cytokine and chemokine production in the lung after influenza infection.** DR1 and B10 mice were infected intranasally with NC or PR8 or were mock-infected with PBS. Mice were sampled 60 h after inoculation. Cytokine and chemokine concentrations in clarified lung homogenates were determined by Multiplex assay. The mean+SE is shown for 5 individual mice per group. * P<0.05, ** P<0.01, *** P<0.001.
(TIFF)

Figure S5 **Ratio of virus-specific IgM ASCs to switched isotype ASCs in DR1 and B10 mice after infection with different viruses.** Data were collected from experiments (presented in figures 3 and 6) in which mice were infected intranasally with NC, PR8, X31, or MHV68, and virus-specific ASC frequencies were determined by ELISpot assay. Ratios, shown for the MedLN on day 8 after infection, were calculated by dividing the IgM ASC frequency by the sum of the IgG1, IgG2b, IgG2c, IgG3, and IgA ASC frequencies. The mean ratio+SE is shown for 4–8 individual mice per group.
(TIFF)

## References

[1] La Gruta NL, Kedzierska K, Stambas J, Doherty PC (2007). A question of self-preservation: immunopathology in influenza virus infection.. Immunol Cell Biol.

[2] Fukuyama S, Kawaoka Y (2011). The pathogenesis of influenza virus infections: the contributions of virus and host factors.. Curr Opin Immunol.

[3] Iwasaki A, Medzhitov R (2010). Regulation of adaptive immunity by the innate immune system.. Science.

[4] Waffarn EE, Baumgarth N (2011). Protective B cell responses to flu–no fluke!. J Immunol.

[5] Lee BO, Rangel-Moreno J, Moyron-Quiroz JE, Hartson L, Makris M (2005). CD4 T cell-independent antibody response promotes resolution of primary influenza infection and helps to prevent reinfection.. J Immunol.

[6] Sangster MY, Riberdy JM, Gonzalez M, Topham DJ, Baumgarth N (2003). An early CD4^+^ T cell-dependent immunoglobulin A response to influenza infection in the absence of key cognate T-B interactions.. J Exp Med.

[7] Goodnow CC, Vinuesa CG, Randall KL, Mackay F, Brink R (2010). Control systems and decision making for antibody production.. Nat Immunol.

[8] Vinuesa CG, Linterman MA, Goodnow CC, Randall KL (2010). T cells and follicular dendritic cells in germinal center B-cell formation and selection.. Immunol Rev.

[9] Sarawar SR, Doherty PC (1994). Concurrent production of interleukin-2, interleukin-10, and gamma interferon in the regional lymph nodes of mice with influenza pneumonia.. J Virol.

[10] Roman E, Miller E, Harmsen A, Wiley J, Von Andrian UH (2002). CD4 effector T cell subsets in the response to influenza: heterogeneity, migration, and function.. J Exp Med.

[11] Peng SL, Szabo SJ, Glimcher LH (2002). T-bet regulates IgG class switching and pathogenic autoantibody production.. Proc Natl Acad Sci U S A.

[12] Stavnezer J (1996). Immunoglobulin class switching.. Curr Opin Immunol.

[13] Richards KA, Chaves FA, Krafcik FR, Topham DJ, Lazarski CA (2007). Direct ex vivo analyses of HLA-DR1 transgenic mice reveal an exceptionally broad pattern of immunodominance in the primary HLA-DR1-restricted CD4 T-cell response to influenza virus hemagglutinin.. J Virol.

[14] Richards KA, Chaves FA, Sant AJ (2009). Infection of HLA-DR1 transgenic mice with a human isolate of influenza a virus (H1N1) primes a diverse CD4 T-cell repertoire that includes CD4 T cells with heterosubtypic cross-reactivity to avian (H5N1) influenza virus.. J Virol.

[15] Joo HM, He Y, Sangster MY (2008). Broad dispersion and lung localization of virus-specific memory B cells induced by influenza pneumonia.. Proc Natl Acad Sci U S A.

[16] Sangster MY, Topham DJ, D'Costa S, Cardin RD, Marion TN (2000). Analysis of the virus-specific and nonspecific B cell response to a persistent B-lymphotropic gammaherpesvirus.. J Immunol.

[17] Woods A, Chen HY, Trumbauer ME, Sirotina A, Cummings R (1994). Human major histocompatibility complex class II-restricted T cell responses in transgenic mice.. J Exp Med.

[18] McDuffie E, Obert L, Chupka J, Sigler R (2006). Detection of cytokine protein expression in mouse lung homogenates using suspension bead array.. J Inflamm (Lond).

[19] Coro ES, Chang WL, Baumgarth N (2006). Type I IFN receptor signals directly stimulate local B cells early following influenza virus infection.. J Immunol.

[20] Shinall SM, Gonzalez-Fernandez M, Noelle RJ, Waldschmidt TJ (2000). Identification of murine germinal center B cell subsets defined by the expression of surface isotypes and differentiation antigens.. J Immunol.

[21] Nayak JL, Richards KA, Chaves FA, Sant AJ (2010). Analyses of the specificity of CD4 T cells during the primary immune response to influenza virus reveals dramatic MHC-linked asymmetries in reactivity to individual viral proteins.. Viral Immunol.

[22] Li X, Vanitha DJ, Joo HM, He Y, Rouse BT (2006). A strategy for selective, CD4^+^ T cell-independent activation of virus-specific memory B cells for limiting dilution analysis.. J Immunol Methods.

[23] Joo HM, He Y, Sundararajan A, Huan L, Sangster MY (2010). Quantitative analysis of influenza virus-specific B cell memory generated by different routes of inactivated virus vaccination.. Vaccine.

[24] Szomolanyi-Tsuda E, Welsh RM (1998). T-cell-independent antiviral antibody responses.. Curr Opin Immunol.

[25] Rosloniec EF, Brand DD, Myers LK, Whittington KB, Gumanovskaya M (1997). An HLA-DR1 transgene confers susceptibility to collagen-induced arthritis elicited with human type II collagen.. J Exp Med.

[26] Divekar AA, Zaiss DM, Lee FE, Liu D, Topham DJ (2006). Protein vaccines induce uncommitted IL-2-secreting human and mouse CD4 T cells, whereas infections induce more IFN-γ-secreting cells.. J Immunol.

[27] Wang X, Mosmann T (2001). In vivo priming of CD4 T cells that produce interleukin (IL)-2 but not IL-4 or interferon (IFN)-γ, and can subsequently differentiate into IL-4- or IFN-γ-secreting cells.. J Exp Med.

[28] Mosmann TR, Cherwinski H, Bond MW, Giedlin MA, Coffman RL (1986). Two types of murine helper T cell clone. I. Definition according to profiles of lymphokine activities and secreted proteins.. J Immunol.

[29] Lipatov AS, Andreansky S, Webby RJ, Hulse DJ, Rehg JE (2005). Pathogenesis of Hong Kong H5N1 influenza virus NS gene reassortants in mice: the role of cytokines and B- and T-cell responses.. J Gen Virol.

[30] Sarawar SR, Sangster M, Coffman RL, Doherty PC (1994). Administration of anti-IFN-γ antibody to β_2_-microglobulin-deficient mice delays influenza virus clearance but does not switch the response to a T helper cell 2 phenotype.. J Immunol.

[31] Graham MB, Dalton DK, Giltinan D, Braciale VL, Stewart TA (1993). Response to influenza infection in mice with a targeted disruption in the interferon γ gene.. J Exp Med.

[32] Reinhardt RL, Liang HE, Locksley RM (2009). Cytokine-secreting follicular T cells shape the antibody repertoire.. Nat Immunol.

[33] King IL, Mohrs M (2009). IL-4-producing CD4^+^ T cells in reactive lymph nodes during helminth infection are T follicular helper cells.. J Exp Med.

[34] Rolf J, Bell SE, Kovesdi D, Janas ML, Soond DR (2010). Phosphoinositide 3-kinase activity in T cells regulates the magnitude of the germinal center reaction.. J Immunol.

[35] Schwickert TA, Victora GD, Fooksman DR, Kamphorst AO, Mugnier MR (2011). A dynamic T cell-limited checkpoint regulates affinity-dependent B cell entry into the germinal center.. J Exp Med.

[36] Chang WL, Coro ES, Rau FC, Xiao Y, Erle DJ (2007). Influenza virus infection causes global respiratory tract B cell response modulation via innate immune signals.. J Immunol.

[37] Liu N, Ohnishi N, Ni L, Akira S, Bacon KB (2003). CpG directly induces T-bet expression and inhibits IgG1 and IgE switching in B cells.. Nat Immunol.

[38] Jegerlehner A, Maurer P, Bessa J, Hinton HJ, Kopf M (2007). TLR9 signaling in B cells determines class switch recombination to IgG2a.. J Immunol.

[39] Stavnezer J, Guikema JE, Schrader CE (2008). Mechanism and regulation of class switch recombination.. Annu Rev Immunol.

[40] Kim SJ, Caton M, Wang C, Khalil M, Zhou ZJ (2008). Increased IL-12 inhibits B cells' differentiation to germinal center cells and promotes differentiation to short-lived plasmablasts.. J Exp Med.

[41] Rothaeusler K, Baumgarth N (2010). B-cell fate decisions following influenza virus infection.. Eur J Immunol.

[42] Schmitz N, Kurrer M, Bachmann MF, Kopf M (2005). Interleukin-1 is responsible for acute lung immunopathology but increases survival of respiratory influenza virus infection.. J Virol.

[43] Arima K, Watanabe N, Hanabuchi S, Chang M, Sun SC (2010). Distinct signal codes generate dendritic cell functional plasticity.. Sci Signal.

[44] Yoo JK, Galligan CL, Virtanen C, Fish EN (2010). Identification of a novel antigen-presenting cell population modulating antiinfluenza type 2 immunity.. J Exp Med.

