# Supplementary figures and images for: Host Differences in Influenza-Specific CD4 T Cell and B Cell Responses Are Modulated by Viral Strain and Route of Immunization
Source: PLoS One. 2012 Mar 23;7(3):e34377. doi: 10.1371/journal.pone.0034377 (PMC3311631; doi:10.1371/journal.pone.0034377)

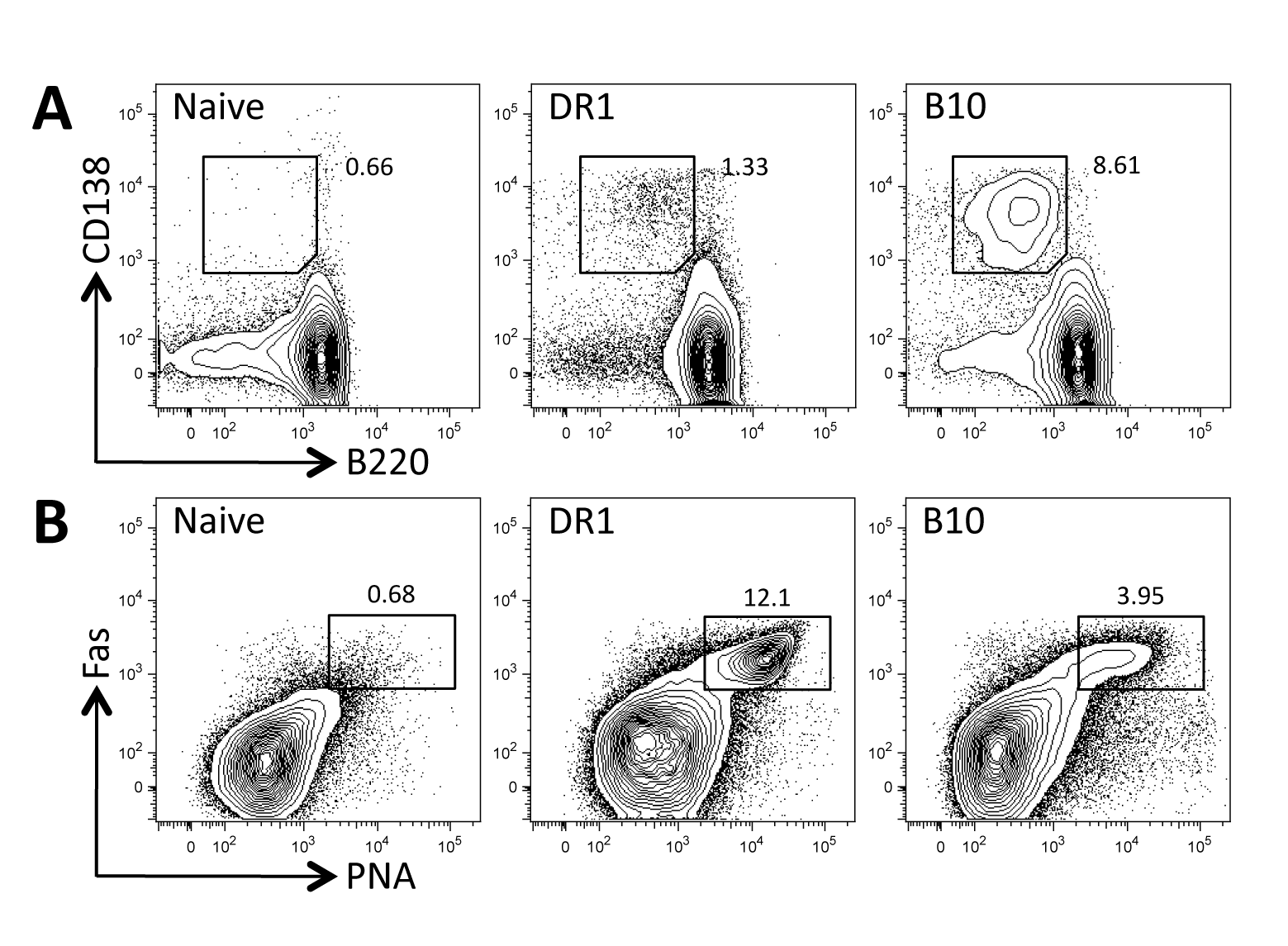

Supplement: Figure S1 — Flow cytometric identification of ASCs and germinal center B cells. MedLN cells were analyzed on day 8 after intranasal NC infection of DR1 and B10 mice. Representative 5% contour plots are shown for individual DR1 and B10 mice after infection and for naïve control mice. Plots depict B220 and CD138 expression after gating on live CD4− CD8− CD19+ cells (A) or PNA-binding and Fas expression after gating on live CD4− CD8− B220+ cells (B). Numbers indicate cell frequencies in the depicted gates identifying ASCs (defined as B220int CD138+) and germinal center B cells (defined as PNA+ Fas+). (TIF) [file pone.0034377.s001.tif]

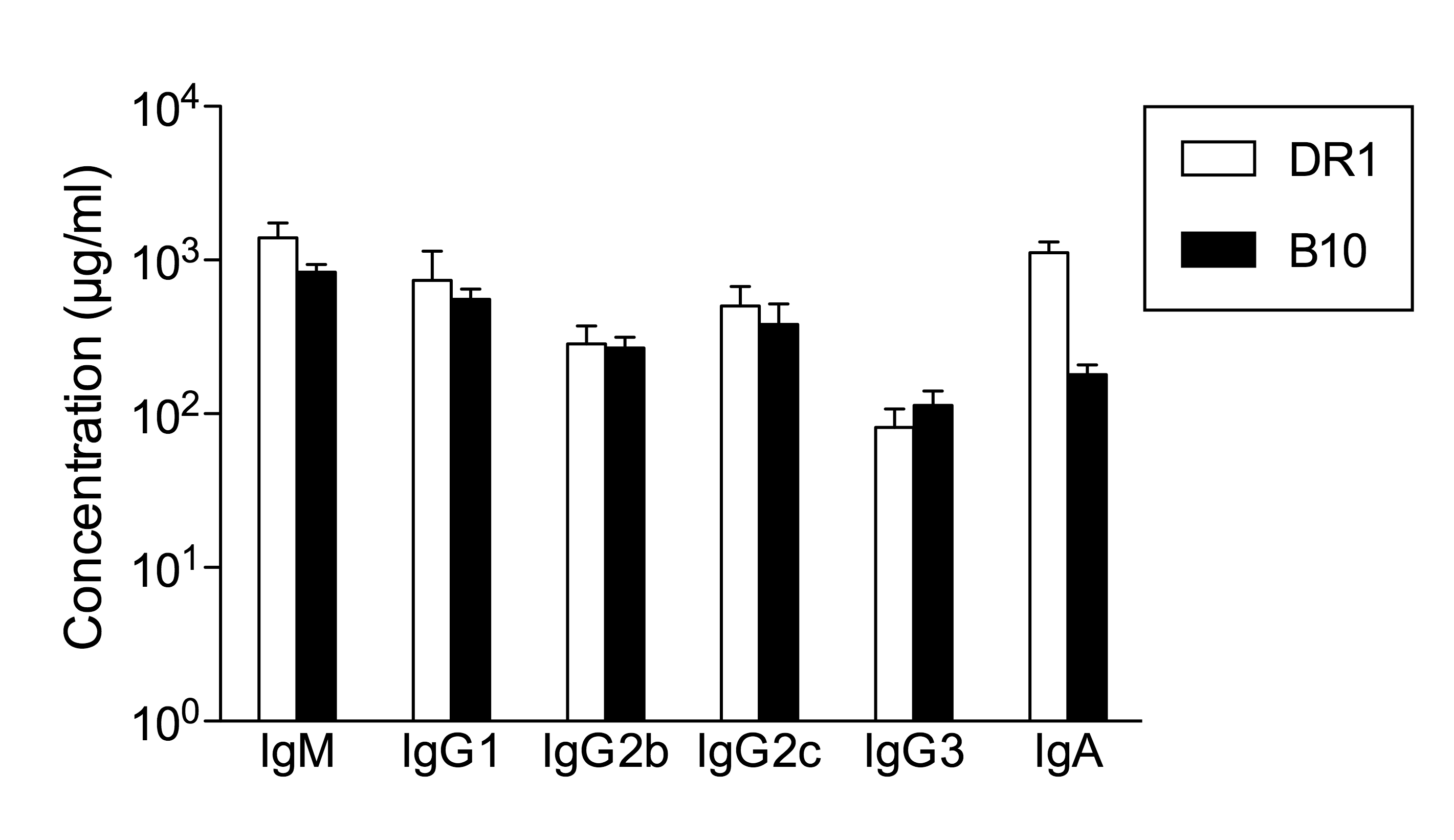

Supplement: Figure S2 — Total serum levels of Ab isotypes in uninfected DR1 and B10 mice. Titers were determined by ELISA and quantified by reference to standards of known concentration. The mean+SE is shown for 5 individual mice per group. (TIFF) [file pone.0034377.s002.tiff]

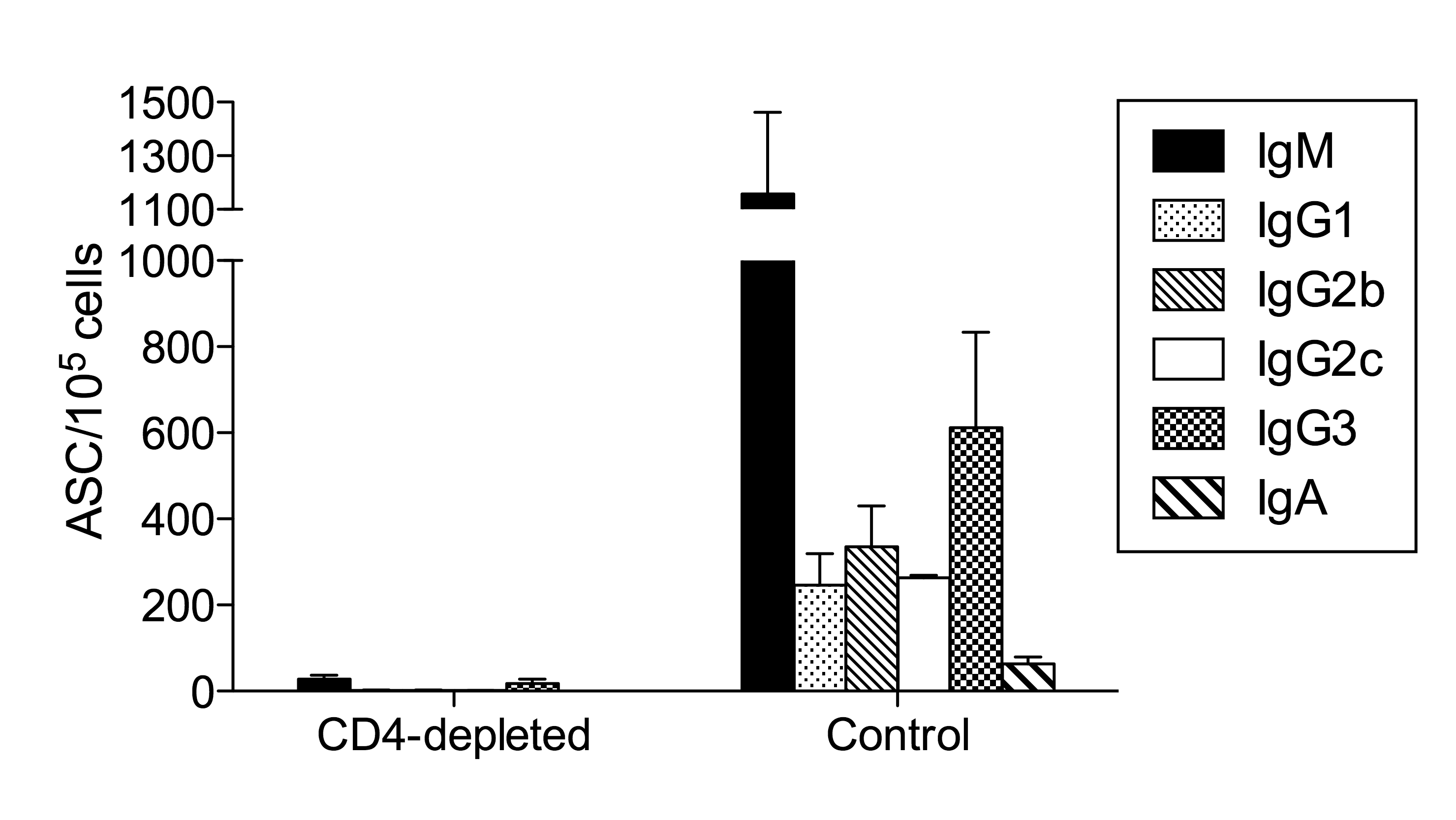

Supplement: Figure S3 — The virus-specific B cell response to PR8 infection in DR1 mice is CD4 T cell-dependent. PR8-specific ASC frequencies in the MedLN of CD4 T cell-depleted DR1 mice and mock-depleted control mice were determined by ELISpot assay on day 8 after infection. The mean+SE is shown for 4 individual mice per group. (TIFF) [file pone.0034377.s003.tiff]

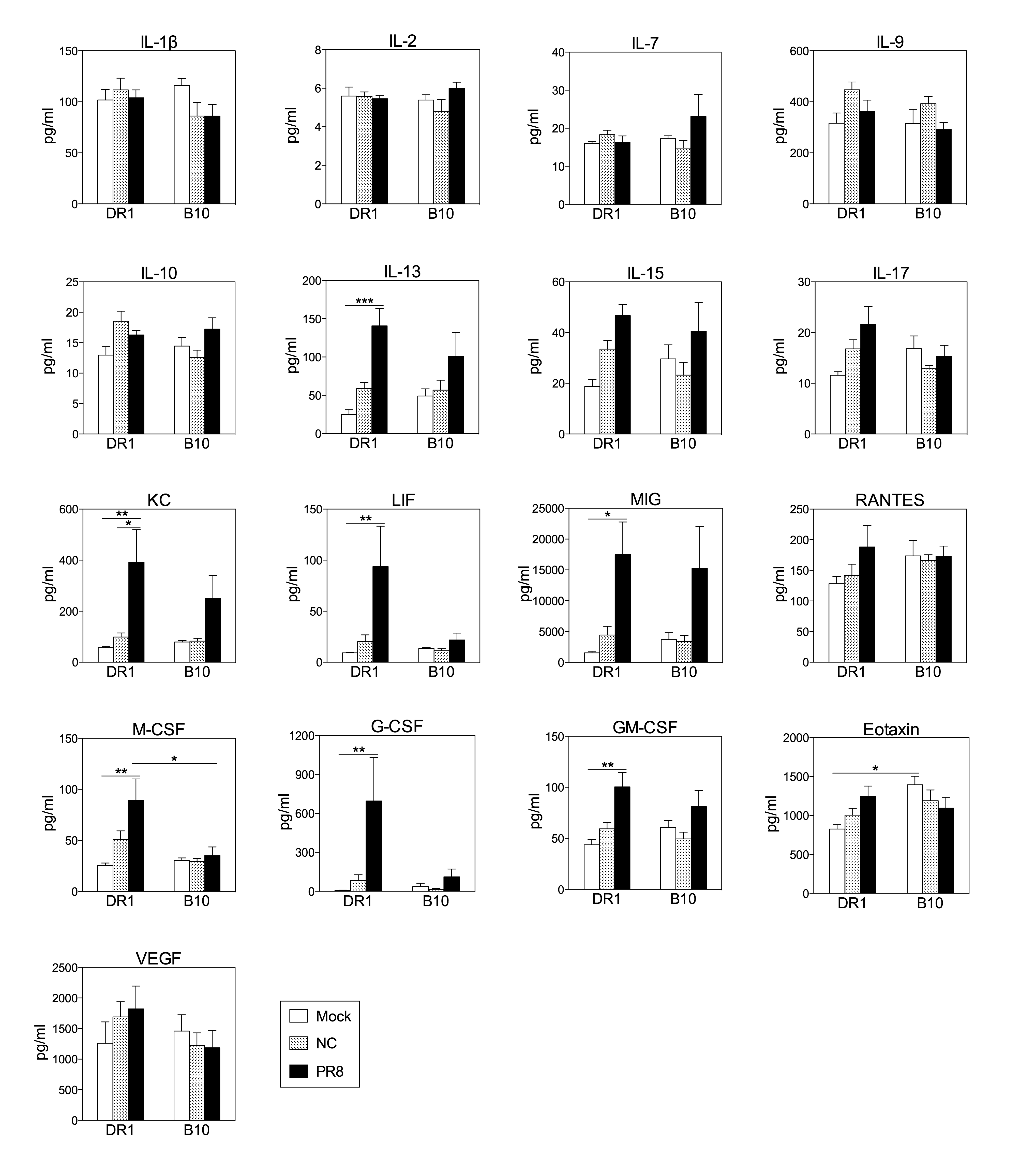

Supplement: Figure S4 — Cytokine and chemokine production in the lung after influenza infection. DR1 and B10 mice were infected intranasally with NC or PR8 or were mock-infected with PBS. Mice were sampled 60 h after inoculation. Cytokine and chemokine concentrations in clarified lung homogenates were determined by Multiplex assay. The mean+SE is shown for 5 individual mice per group. * P<0.05, ** P<0.01, *** P<0.001. (TIFF) [file pone.0034377.s004.tiff]

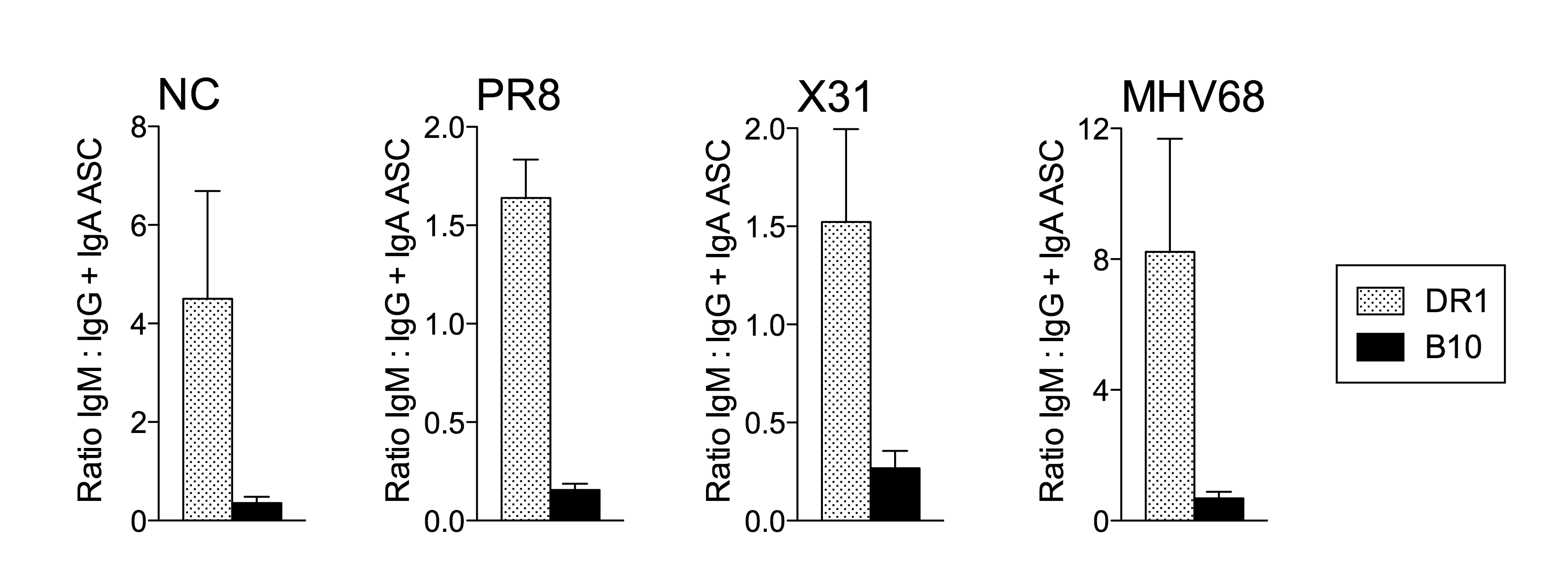

Supplement: Figure S5 — Ratio of virus-specific IgM ASCs to switched isotype ASCs in DR1 and B10 mice after infection with different viruses. Data were collected from experiments (presented in figures 3 and 6) in which mice were infected intranasally with NC, PR8, X31, or MHV68, and virus-specific ASC frequencies were determined by ELISpot assay. Ratios, shown for the MedLN on day 8 after infection, were calculated by dividing the IgM ASC frequency by the sum of the IgG1, IgG2b, IgG2c, IgG3, and IgA ASC frequencies. The mean ratio+SE is shown for 4–8 individual mice per group. (TIFF) [file pone.0034377.s005.tiff]
